# Fabrication and Characterization of Binary Ti-Al and Ti-Si Thin-Film Metallic Glasses

**DOI:** 10.3390/ma19040802

**Published:** 2026-02-19

**Authors:** Vidžaja Knap, Petr Vlcak, Margit Žaloudková, Tomáš Horažďovský, Jan Drahokoupil, Ján Sovík, Jaroslav Fojt, Jaroslav Lukeš, Vojtěch Smola, Zdeněk Weiss

**Affiliations:** 1 Department of Physics, Faculty of Mechanical Engineering, Czech Technical University in Prague, Technicka 4, 166 07 Prague, Czech Republic; 2 Department of Composites and Carbon Materials, Institute of Rock Structure and Mechanics, Czech Academy of Sciences, V Holešovičkách 41, 182 09 Prague, Czech Republic; zaloudkova@irsm.cas.cz; 3 Research Centre, University of Žilina, Univerzitná 8215/1, 010 26 Žilina, Slovakia; 4 Department of Metals and Corrosion Engineering, University of Chemistry and Technology in Prague, Technicka 5, 166 28 Prague, Czech Republic; 5 Department of Mechanics, Biomechanics and Mechatronics, Faculty of Mechanical Engineering, Czech Technical University in Prague, Technicka 4, 166 07 Prague, Czech Republic; 6 FZU-Institute of Physics of the Czech Academy of Sciences, Na Slovance 2, 182 00 Prague, Czech Republic; weissz@fzu.cz

**Keywords:** thin-film metallic glasses, Ti-Si alloys, Ti-Al alloys, corrosion resistance

## Abstract

This study investigates the fabrication and characterization of binary Ti-Si and Ti-Al thin-film metallic glasses (TFMGs) deposited via electron beam evaporation on cp Ti and Si substrates. X-ray diffraction confirmed the amorphous structure of the Ti_89_Si_11_ and Ti_55_Al_45_ thin films. AFM revealed differences in surface roughness, with Ti_89_Si_11_ exhibiting a smoother surface (Ra = 0.9 nm) than Ti_55_Al_45_ (Ra = 1.6 nm), likely due to differences in atomic size mismatch, heat of mixing, and potential oxidation effects. Electrochemical tests in Hank’s solution demonstrated the superior corrosion resistance of Ti_89_Si_11_, which had the lowest i_corr_ (0.123 µA/cm^2^) and widest passive region compared to Ti_55_Al_45_ and reference materials (cp Ti and SS316L). Mechanical properties revealed that both TFMGs exhibit higher indentation hardness and comparable reduced elastic modulus to cp Ti, with Ti_55_Al_45_ showing the highest hardness (5.93 ± 0.37 GPa). These findings highlight the potential of Ti-Si and Ti-Al TFMGs as high-performance materials for biomedical coatings.

## 1. Introduction

Metallic glasses are gaining attention as high-performance materials due to their unique combination of mechanical, chemical, and surface properties. These qualities arise from their amorphous atomic structure, which lacks grain boundaries and crystalline defects, making them promising candidates for engineering and biomedical applications. For coatings on biomedical implants, particularly orthopedic and dental ones, metallic glasses address key challenges such as corrosion in physiological environments and mechanical compatibility with bone tissue [1,2]. To address these issues, various surface modification techniques have been explored, including physical vapor deposition (PVD) of hard ceramic coatings (e.g., TiN, TiCN) and diamond-like carbon (DLC) films [3,4].

However, these polycrystalline or carbon-based coatings often face challenges related to adhesion, brittleness, or structural defects [3,5]. Conventional biomaterials, like Fe-based alloys (e.g., 316L stainless steel), suffer from corrosion, releasing toxic ions and causing stress-shielding due to elastic modulus mismatches with bone [6,7]. While titanium-based alloys provide excellent corrosion resistance, their lower hardness and wear resistance remain limitations [8,9]. In contrast, metallic glasses exhibit high strength, reduced modulus mismatch, superior corrosion resistance, and enhanced hardness—properties essential for reducing toxicity and wear in the body [10,11]. Among these, Ti-based metallic glasses are particularly attractive for their excellent biocompatibility and ability to integrate with bone [12]. Despite these advantages, metallic glasses often require complex multi-element compositions to enhance glass-forming ability and prevent crystallization [13]. Fundamental studies on the metallurgy of metallic glasses emphasize that high glass-forming ability is typically associated with deep eutectics, significant atomic size mismatch (>12%), and negative heat at mixing among constituent elements [14,15,16,17]. These empirical rules guide the selection of appropriate alloy systems to suppress nucleation and ensure amorphous structure retention during deposition.

Thin-film metallic glasses (TFMGs) provide a promising solution to the challenges of producing bulk metallic glasses with complex compositions [18]. Notably, binary TFMGs remain relatively understudied, despite their potential to simplify alloy design, reduce manufacturing costs, and improve reproducibility compared to multi-component systems. To leverage these advantages, this work focuses on two binary TFMG systems—Ti-Si and Ti-Al—chosen for their anticipated benefits in biocompatibility, corrosion resistance, and mechanical performance as potential implant coatings. Although preliminary studies hint at their suitability for enhancing such biomedical applications, these compositions are largely unexplored [19,20,21]. Specifically, Ti-Si appears to encourage hydroxyapatite formation, a key factor in promoting osseointegration for orthopedic and dental implants [22], while emerging research on Ti-Al suggests antibacterial properties that could be advantageous in implant-related medical environments [23].

The compositions Ti_89_Si_11_ and Ti_55_Al_45_ were selected based on prior reports of glass-forming ability in binary Ti-Si and Ti-Al systems [19,20,21], followed by experimental screening to identify compositions balancing amorphization with functional properties. Furthermore, for the Ti-Si alloy, the chosen Si content avoids excessive Si (>15 at.%), which could increase the elastic modulus [24]. This balance ensures modulus values closer to bone (∼30 GPa), mitigating stress shielding [13]. To the best of our knowledge, this study represents one of the first systematic comparative investigations of binary Ti–Si and Ti–Al thin-film metallic glass coatings produced by electron beam evaporation, focusing on their structure, surface morphology, corrosion behavior in Hank’s solution, and nanoindentation-derived mechanical properties. By benchmarking the coatings against commercially pure titanium and SS316L, this work aims to clarify the potential of simplified binary TFMGs to combine corrosion protection with mechanical properties relevant to biomedical implant coatings. Moreover, by deliberately employing binary systems, this study provides insight into structure–property relationships while highlighting a cost-effective and scalable alternative to complex multi-component metallic glass coatings.

## 2. Experimental Methods

Ti-Si and Ti-Al coatings were synthesized on both polished commercially pure titanium (grade II, cp-Ti) and silicon wafer substrates. Ti samples (5 mm in height and 14 mm in diameter) were ground with silicon carbide abrasive papers (P320–P1200) and polished using diamond pastes (9 μm, 3 μm and 1 μm) and colloidal silica suspension (0.02 μm). Silicon wafers were cleaned by ultrasonic degreasing in isopropyl alcohol for 20 min.

The coatings, approximately 400 nm thick, were deposited in a vacuum chamber using simultaneous electron beam evaporation from two separate targets—titanium and silicon for Ti-Si coatings and titanium and aluminum for Ti-Al coatings. The samples were mounted on a rotary manipulator, ensuring uniform deposition. The deposition rates were monitored using a quartz crystal microbalance and regulated by adjusting the electron beam power. The chamber pressure during deposition was maintained at approximately 8 × 10^−4^ Pa.

The composition of the coatings was analyzed via glow discharge emission spectroscopy (GDOES) using the GDA750HR spectrometer (Spectruma Analytik GmbH, Hof, Germany). Emission lines used for Ti, Si, and Al were Ti I (499.864 nm), Si I (288.158 nm), and Al I (396.152 nm), respectively. Quantitative depth profiles of the coatings were measured using a sputter rate-corrected calibration [25], established specifically for the Ti-Al-Si system and based on certified reference materials of various Ti- and AlSi alloys and steels. Two samples with nominal compositions of Ti_55_Al_45_ and Ti_89_Si_11_ were chosen for further examination as described below.

Surface chemical analysis was performed by X-ray photoelectron spectroscopy (XPS) using an ESCAprobe P spectrometer (Omicron Nanotechnology Ltd., London, UK) equipped with a monochromatic Al Kα radiation source (E = 1486.7 eV). The base pressure in the analysis chamber was maintained at 2 × 10^−8^ Pa. Spectra were acquired with an energy step of 0.05 eV, and the binding energy scale was calibrated to the adventitious carbon C 1s peak at 285.0 eV. Data processing involved background subtraction using the non-linear iterative Shirley method, followed by peak fitting using a least-squares algorithm with mixed Gaussian/Lorentzian functions. Specifically, a symmetric peak shape (GL, 30) was used for oxide components, while an asymmetric line shape [LA (1.1,5,7)] was applied for metallic states, in accordance with the NIST X-ray Photoelectron Spectroscopy Database [26]. The oxide layer thickness was estimated based on the intensity ratio of the oxide and metallic components using the method described by Strohmeier [27] and Carlson [28]. It should be noted that these thickness calculations assume a pure oxide overlayer for each respective metal element.

X-ray diffraction (XRD) measurements were performed on a X’Pert PRO (PANalytical, Malvern, UK) horizontal powder diffractometer equipped with a Co anode (λ = 0.1789 nm). Parallel beam geometry was used to enhance the contribution of the surface layer—a Göbel mirror in the primary beam and a parallel plate collimator with acceptance 0.09 in the diffracted beam. The angle of incidence was fixed at 3°.

Atomic force microscopy (AFM) measurements were performed using a Nanowizard3 (JPK, Berlin, Germany) in contact mode in air, with a scan area of 2 × 2 μm and an image resolution of 512 pixels. A CONTV-A cantilever (Bruker, Camarillo, CA, USA) with a spring constant of 0.2 N/m was used, and images were flattened using plane fitting to correct for sample tilt. Surface topography and roughness parameters, specifically the arithmetic mean roughness (Ra) and root-mean-square roughness (Rq), were determined by averaging results from ten distinct scanned areas for each sample type.

Scanning electron microscopy (SEM) (ThermoFisher Scientific, Waltham, MA, USA) was used to analyze the cross-sectional and surface morphology of the samples. Samples for cross-section analysis were mounted on an aluminum stub using carbon tape and coated with a 12.6 nm platinum layer in an argon atmosphere using a Leica EM ACE600 sputter coater (Specion s.r.o., Prague, Czech Republic). Imaging was performed using an Apreo S LoVac scanning electron microscope (ThermoFisher Scientific, Waltham, MA, USA) in high vacuum mode, employing Everhart–Thornley (ETD) and T1/T2 Trinity in-lens detectors.

Electrochemical performance was evaluated in Hank’s solution at 37 °C using a standard three-electrode configuration with a potentiostat VSP-300 (Biologic, Seyssinet-Pariset, France). A saturated calomel electrode (SCE) served as the reference electrode, and a platinum (Pt) mesh acted as the counter electrode. Potentiodynamic polarization (PDP) tests were conducted by sweeping the potential from −250 mV relative to the open circuit potential (OCP) up to 2 V vs. OCP at a scan rate of 1 mV s^−1^. Polarization data were analyzed using EC Lab 11.50 software.

The mechanical properties were measured using a Hysitron TI 950 TriboIndenter (Bruker, Billerica, MA, USA) with a Performech II controller (Bruker, Billerica, MA, USA) and a Berkovich diamond probe (Bruker, Billerica, MA, USA). Testing involved 9 indents per sample, spatially distributed to avoid interaction effects. At each indent location, the protocol consisted of 15 cycles of loading and 50% unloading, with loads progressively increasing from 200 µN to 1 mN.

## 3. Results and Discussion

**Figure 1:**
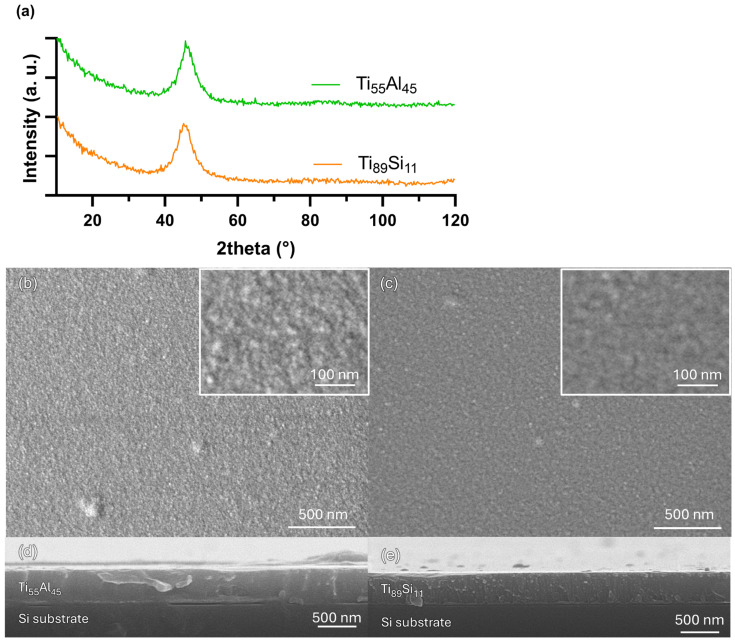
(**a**) XRD patterns of the as-deposited TFMGs. SEM micrographs of amorphous thin films: (**b**,**c**) surface morphology of Ti_55_Al_45_ and Ti_89_Si_11_, respectively, and (**d**,**e**) cross-sectional morphology of Ti_55_Al_45_ and Ti_89_Si_11_, respectively.

X-ray diffraction (XRD) analysis was performed on the Ti_89_Si_11_ and Ti_55_Al_45_ TFMGs, with patterns shown in Figure 1a. Both spectra exhibit a broad amorphous peak centered in the 2θ range of 40–50°. The absence of sharp Bragg peaks confirms the lack of long-range crystalline order in both samples. The full width at half maximum (FWHM) for these maxima was determined as 4.76° for Ti_55_Al_45_ and 5.09° for Ti_89_Si_11_.

Surface and cross-sectional morphologies of the thin films were analyzed using scanning electron microscopy (SEM), as shown in Figure 1b–e. The surface morphologies (Figure 1b,c) appear dense and relatively featureless at this magnification, crucially exhibiting no discernible crystalline grains, which is consistent with the amorphous structure confirmed by XRD. High-magnification observations reveal a remarkably homogeneous surface texture, devoid of granular boundaries, segregations, or pinholes typically associated with crystalline thin films. This featureless morphology is characteristic of the single-phase amorphous state, indicating that the rapid quenching rate achieved during electron beam evaporation effectively suppressed atomic diffusion and crystallization. Furthermore, the cross-sectional SEM images (Figure 1d,e) reveal dense and continuous layers, free from obvious voids or columnar structures often seen in crystalline PVD coatings. This suggests a layer-by-layer growth mode with high adatom mobility, ensuring excellent coating integrity. The film thickness measured from these micrographs is approximately 400 nm, consistent with the target deposition value.

**Figure 2:**
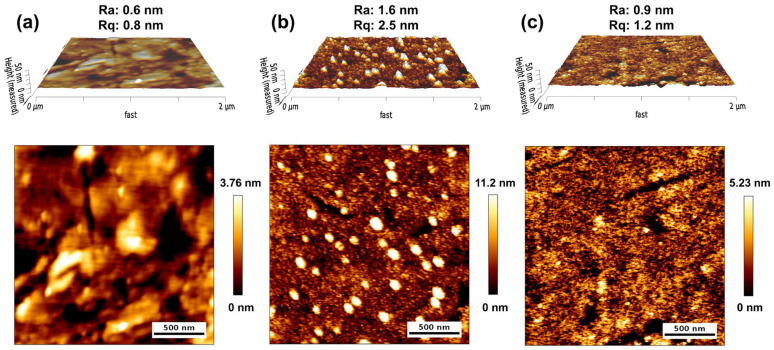
AFM images of (**a**) cp Ti polished sample; (**b**) Ti_55_Al_45_; and (**c**) Ti_89_Si_11_ TFMGs.

**Figure 3:**
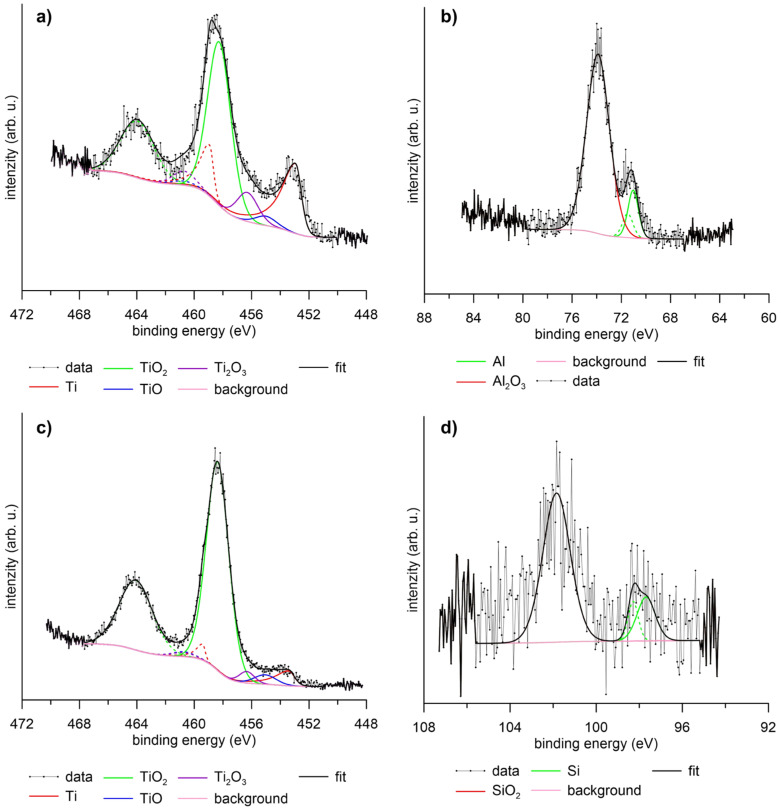
High-resolution XPS spectra of the TFMG surfaces: (**a**) Ti 2p and (**b**) Al 2p regions for Ti_55_Al_45_; (**c**) Ti 2p and (**d**) Si 2p regions for Ti_89_Si_11_.

Atomic force microscopy (AFM) was employed to examine the topography and quantify surface roughness of the polished cp Ti substrate and the Ti-Al and Ti-Si TFMGs deposited on polished cp Ti (Figure 2). The polished cp Ti surface (Figure 2a) exhibited a low roughness (Ra = 0.6 nm, Rq = 0.8 nm). The Ti_89_Si_11_ sample (Figure 2c) displayed a very smooth surface with fine textural features (Ra = 0.9 nm, Rq = 1.2 nm), consistent with its amorphous structure and likely aided by the strong Ti-Si chemical affinity (ΔHmix = −66 kJ/mol) promoting uniform, dense packing during growth [29,30]. In stark contrast, the Ti_55_Al_45_ sample (Figure 2b) exhibited significantly higher roughness with distinct, somewhat nodular features (Ra = 1.6 nm, Rq = 2.5 nm). This pronounced difference in topography is attributed to a combination of factors inherent to the Ti-Al system and its interaction with the environment. Firstly, during deposition, the weaker Ti-Al chemical interaction (ΔHmix = −30 kJ/mol) and minimal atomic size mismatch (2.7%), compared to Ti-Si (20.4% mismatch) [29], may facilitate greater surface atom mobility or nanoscale compositional fluctuations, leading to an intrinsically coarser, nodular growth morphology. Secondly, upon exposure to air, the high concentration of reactive aluminum (45 at.%) inevitably leads to surface oxidation. This process, potentially involving non-uniform formation of Al_2_O_3_ alongside titanium oxides, likely further enhances the roughness established during deposition. To elucidate the chemical origin of the surface topography, XPS analysis was performed (Figure 3). The Ti 2p and Al 2p spectra for Ti_55_Al_45_ (Figure 3a,b) confirm the coexistence of TiO_2_ and Al_2_O_3_ on the surface. Quantitative analysis based on the Strohmeier and Carlson method [27,28] indicates a relatively thin mixed-oxide layer, consisting of approximately 3.6 nm of titanium oxide and 1.2 nm of aluminum oxide. The concurrent growth of these distinct oxide phases (TiO_2_ and Al_2_O_3_), which possess different molar volumes, likely induces local lattice strains and heterogeneous oxide growth. This chemical heterogeneity directly contributes to the higher roughness (Ra = 1.6 nm) and nodular morphology observed in AFM measurements compared to the uniform Ti-Si coating.

**Figure 4:**
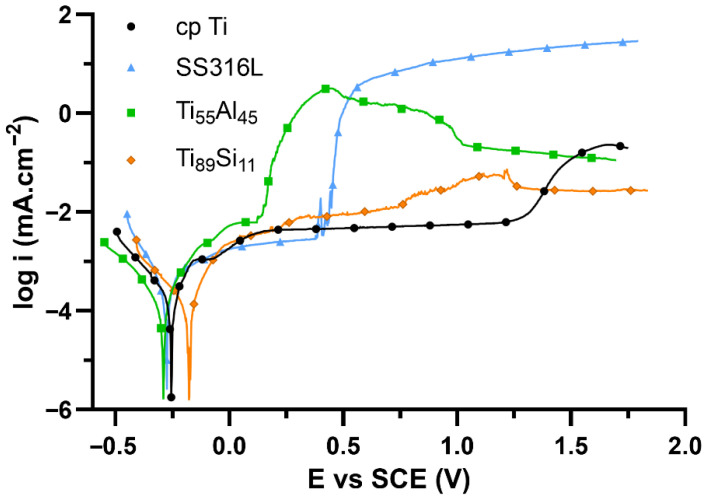
Potentiodynamic polarization curves of Ti_55_Al_45_, Ti_89_Si_11_, SS316L and cp Ti.

Figure 4 displays the polarization curves, while Table 1 summarizes the electrochemical parameters of the tested materials and Ti-based TFMGs from other studies. Cp Ti and SS316L are included as benchmark materials for corrosion resistance comparison. The i_corr_ values of both TFMGs are lower than those of cp Ti (0.202 µA/cm^2^) and SS316L (0.305 µA/cm^2^), highlighting their superior corrosion resistance. Among the TFMGs, Ti_89_Si_11_ demonstrates the best corrosion performance, with the lowest i_corr_ (0.123 µA/cm^2^) and a relatively positive E_corr_ (−179.6 mV). Conversely, despite its amorphous structure, Ti_55_Al_45_ shows a more negative E_corr_ (−291.7 mV) and a slightly higher i_corr_ (0.175 µA/cm^2^), indicating less favorable corrosion resistance.

In comparison with studies that prepared multi-element Ti-based TFMGs (Table 1) [31,32], binary coatings exhibit lower corrosion current densities than the Ti_60_Nb_15_Zr_10_Si_15_ alloy (i_corr_ = 0.220 µA/cm^2^) despite having more negative E_corr_ values. Relative to ternary Ti-Zr-Si variants (e.g., Ti_47_Zr_41_Si_12_, i_corr_ = 0.164 µA/cm^2^; Ti_58_Zr_33_Si_9_, i_corr_ = 0.086 µA/cm^2^), the binary TFMGs display comparable i_corr_ values but significantly less negative E_corr_ (−179.6 mV vs. −500 mV for Ti_58_Zr_33_Si_9_). To ensure the reliability and reproducibility of the electrochemical measurements, each polarization test was repeated at least three times on independently prepared samples. Comparable polarization behavior was observed in all measurements. The corrosion potential (E_corr_) and corrosion current density (i_corr_) exhibited standard deviations below 10%, confirming good measurement reproducibility and a high degree of coating homogeneity.

In terms of passivation, the SS316L exhibits a relatively stable passive region from approximately −0.1 to 0.4 V vs. the saturated calomel electrode (SCE) while the cp Ti shows a wide passive region spanning over 1.4 V range with transpassive behavior starting at around 1.3 V vs. SCE. The Ti_55_Al_45_ sample exhibits a relatively narrow passive region, with the passive film breaking down at around 0.15 V vs. SCE. This suggests early onset of transpassive behavior, which can be attributed to the thermodynamic instability of the aluminum-rich oxide layer in chloride-rich environments. Although Al can form a passive Al_2_O_3_ film, it is less stable than TiO_2_ in Hank’s solution, where preferential dissolution of Al sites creates local defects leading to the observed lower breakdown potential [13]. However, at higher potentials (from approximately 1 V vs. SCE onward), the current density begins to decrease, indicating a re-passivation process. Notably, at around 1.75 V vs. SCE, the current density is lower than that of cp Ti. In the Ti_89_Si_11_ sample, the current density decreases at higher potentials, suggesting the formation of a highly stable oxide layer. This stability was structurally verified by XPS analysis (Figure 3c,d). The Si 2p spectrum reveals a distinct peak at ~102 eV, corresponding to oxidized silicon (SiO_2_), confirming that silicon is chemically incorporated into the passive film. Most notably, the calculated thickness of the titanium oxide layer in Ti_89_Si_11_ is approximately 7.9 nm, which is significantly thicker than the oxide layer formed on Ti_55_Al_45_ (~4.8 nm total thickness). This thicker, silicate-incorporated amorphous network acts as a robust barrier against Cl^−^ ion ingress, effectively extending the passive region compared to the thinner, Al-containing film [22]. This contrast in stability is visually confirmed by post-corrosion SEM analysis (Figure S1), where Ti_89_Si_11_ retained a relatively pristine morphology, whereas Ti_55_Al_45_ exhibited surface changes indicative of passive film degradation. It is also noteworthy that the passive ranges achieved by these binary films are similar to those observed in ternary Ti-Zr-Si alloys (e.g., Ti_58_Zr_33_Si_9_, E_pit_ > 2.0 V), with passivity extending beyond 1.8 V. This further supports the potential of simpler compositions for effective corrosion resistance, as the binary Ti-Si system matches or exceeds the performance of more compositionally complex counterparts.

A significant consideration for the Ti_55_Al_45_ TFMG is its high aluminum content (45 at.%), raising potential questions about biocompatibility related to Al ion release. To provide context regarding the total potential reservoir, the amount of aluminum present in the ~400 nm thick coating on a representative large orthopedic implant surface (e.g., a femoral stem with an estimated area of ~200 cm^2^) can be calculated to be less than 10 mg. While this calculation demonstrates that the total amount of aluminum is finite and relatively small, especially considering the Tolerable Weekly Intake (TWI) for aluminum established by EFSA is 1 mg/kg body weight [33], the critical factor determining long-term biological response is primarily the rate and chemical form of ion release into the surrounding physiological environment, rather than the total bulk amount present. The electrochemical results presented here (Figure 4, Table 1) are key in this regard. They indicate that the Ti_55_Al_45_ TFMG forms a passive layer in Hank’s solution, exhibiting a corrosion current density (i_corr_) lower than both cp Ti and SS316L reference materials. This suggests that despite the high Al concentration, the inherent corrosion resistance provided by the passive film formation significantly limits the rate of ion dissolution under these conditions. Although the passivation of Ti_55_Al_45_ appeared less stable at lower potentials compared to Ti_89_Si_11_, the overall low corrosion current implies a correspondingly low rate of Al ion release. However, while these electrochemical findings are promising, direct biological evaluations, such as in vitro cytotoxicity assays, are essential to directly assess the cellular response and confirm the material’s suitability for biomedical applications.

**Figure 5:**
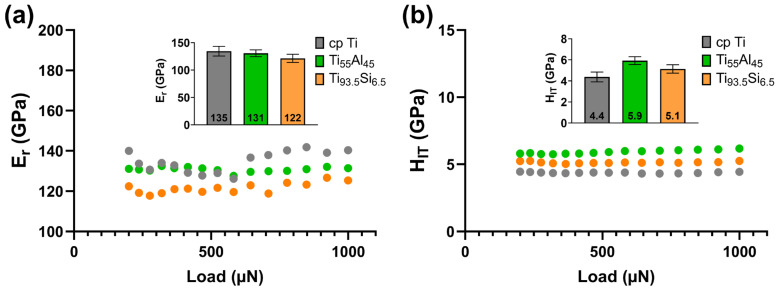
(**a**) Reduced elastic modulus (E_r_) and (**b**) indentation hardness (H_IT_) as a function of depth of contact of the indenter with the sample (h_c_) for both TFMGs and cp Ti. Insets show the mean values with error bars.

The reduced elastic modulus (E_r_) and indentation hardness (H_IT_) data for the tested Ti-based TFMGs are shown in Figure 5. The mean E_r_ for cp Ti was 134.7 ± 8.8 GPa, while TFMGs exhibited slightly lower values: 130.8 ± 6.3 GPa for Ti_55_Al_45_ and 121.6 ± 7.4 GPa for Ti_89_Si_11_. This reduction is likely due to differences in atomic bonding and the disordered structural organization characteristic of their amorphous state [34]. The H_IT_ measurements reveal distinct differences among the materials. The reference cp Ti exhibited a mean indentation hardness of 4.38 ± 0.47 GPa, consistent with expectations for polished titanium. In contrast, the TFMGs demonstrated significantly higher H_IT_ values, with 5.93 ± 0.37 for Ti_55_Al_45_ and 5.14 ± 0.40 GPa for Ti_89_Si_11_. This increase in H_IT_ can be attributed to the dense atomic packing and significant interaction of atoms within the amorphous matrix, which reduces the free volume and enhances the material’s resistance to deformation [35].

## 4. Conclusions

This study demonstrates that Ti_89_Si_11_ and Ti_55_Al_45_ thin-film metallic glasses (TFMGs) deposited via electron beam evaporation exhibit an amorphous structure and notable improvements over pure titanium. Both TFMGs displayed higher indentation hardness—Ti_55_Al_45_ was approximately 35% harder than cp Ti, whereas Ti_89_Si_11_ was around 17% harder. Notably, when compared to more complex ternary and quaternary TFMGs, the binary films not only maintained competitive corrosion current densities—with Ti_89_Si_11_ showing about 40% lower i_corr_ than cp Ti—but also achieved passive ranges similar to those of multi-element systems, despite exhibiting more negative corrosion potentials in some cases. Overall, Ti_89_Si_11_ provided the most advantageous performance by combining improved hardness, a roughly 10% lower reduced elastic modulus, superior corrosion resistance, and the widest passive region in Hank’s solution. These enhancements suggest that the simpler Ti–Si binary TFMGs are especially appealing as potential coatings for orthopedic and dental implants, effectively reducing corrosion-related risks and mitigating stress shielding while simplifying alloy design. Future work will focus on in vitro biological assessments to further validate the biocompatibility suggested by the electrochemical results.

## Figures and Tables

**Table 1:** Electrochemical parameters of Ti-based TFMGs versus reference materials.

	i_corr_ (µA/cm^2^)	i_pass_ (µA/cm^2^)	E_corr_ (mV)	E_pit._ (V)	Ref.
cp Ti	0.202	4.9	−257.4	1.31	
SS316L	0.305	2.0	−276.7	0.38	
Ti_55_Al_45_	0.175	158.5	−291.7	>1.8	
Ti_89_Si_11_	0.123	26.9	−179.6	>1.8	
Ti_60_Nb_15_Zr_10_Si_15_	0.220	-	41.8	-	[31]
Ti_47_Zr_41_Si_12_	0.164	20.7	−530	>2.0	[32]
Ti_58_Zr_33_Si_9_	0.086	4.2	−500	>2.0	[32]
Ti_66_Zr_25_Si_9_	0.081	5.5	−460	>2.0	[32]
Ti_75_Zr_19_Si_6_	0.296	16.9	−370	>2.0	[32]

## Data Availability

The original contributions presented in this study are included in the article/Supplementary Material. Further inquiries can be directed to the corresponding author.

## Supplementary Materials

The following supporting information can be downloaded at https://www.mdpi.com/1996-1944/19/4/802/s1, Figure S1: SEM micrographs of (a) Ti55Al45 and (b) Ti89Si11 thin film metallic glasses after potentiodynamic polarization tests in Hank’s solution.
