# Supplementary material for: Fabrication and Characterization of Binary Ti-Al and Ti-Si Thin-Film Metallic Glasses
Source: Materials (Basel). 2026 Feb 19;19(4):802. doi: 10.3390/ma19040802 (PMC13319933; doi:10.3390/ma19040802)
Supplement: Supplementary file 1 [file materials-19-00802-s001.zip › materials-4114990-supplementary.pdf]

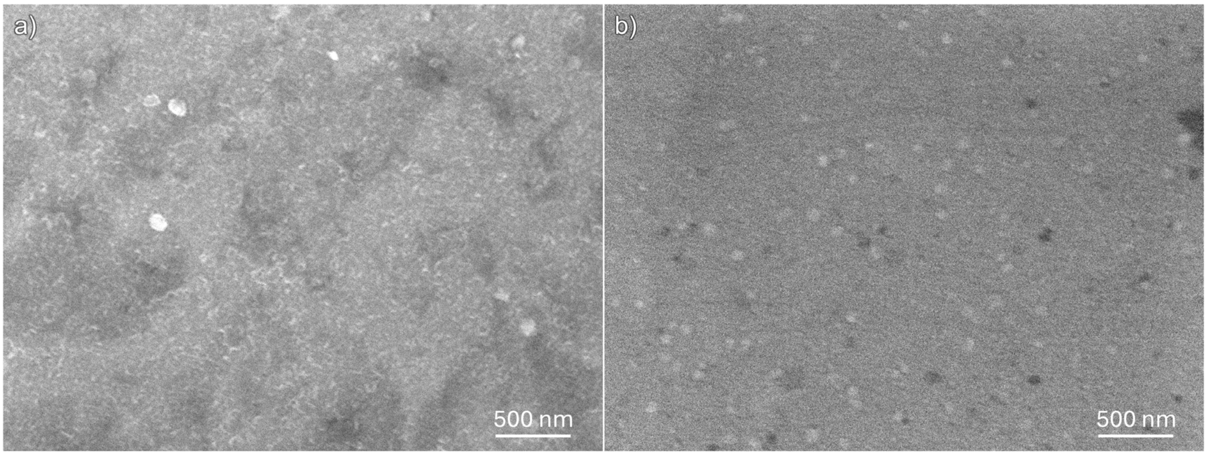

Figure S1. SEM micrographs of the (a) $Ti_{55}Al_{45}$ and (b) $Ti_{89}Si_{11}$ thin film metallic glasses after potentiodynamic polarization tests in Hank's solution.

The $Ti_{55}Al_{45}$ surface (a) exhibits noticeable surface roughening and texture changes, indicative of the formation of less stable oxidation products corresponding to the breakdown observed in electrochemical tests. In contrast, the $Ti_{89}Si_{11}$ surface (b) retains a comparatively smooth and dense morphology without evidence of severe localized corrosion (pitting) or coating delamination, confirming the superior stability of the Si-stabilized passive film.
